# Near-Infrared Light-Triggered Photodynamic Therapy and Apoptosis Using Upconversion Nanoparticles With Dual Photosensitizers

**DOI:** 10.3389/fbioe.2020.00275

**Published:** 2020-04-16

**Authors:** Song Yeul Lee, Ruda Lee, Eunha Kim, Sanghee Lee, Yong Il Park

**Affiliations:** ^1^School of Chemical Engineering, Chonnam National University, Gwangju, South Korea; ^2^International Research Organization for Advanced Science and Technologyn, Kumamoto University, Kumamoto, Japan; ^3^Department of Molecular Science and Technology, Ajou University, Suwon, South Korea; ^4^Center for Neuro-Medicine, Brain Science Institute, Korea Institute of Science and Technology, Seoul, South Korea

**Keywords:** near-infrared, upconversion, nanoparticle, photodynamic therapy, apoptosis

## Abstract

Elucidation of upconversion nanoparticles (UCNPs) that can be excited by near-infrared (NIR) light is an interesting topic in the field of photodynamic therapy (PDT). However, the PDT efficiency of conventional UCNPs is limited due to the low quantum yield and overheating effect of the 980 nm light source. In this study, a light source with a wavelength of 808 nm was used as an excitation source for Nd-doped UCNPs to solve the overheating effect. UCNPs with a core@shell structure (NaYF_4_:Yb,Er,Nd@NaYF_4_:Yb,Nd) were synthesized to increase the upconversion emission efficiency. Dual-color emitting Er-doped UCNPs and dual photosensitizers (Chlorin e6 and Rose Bengal) were used for enhanced PDT. Each photosensitizer could absorb red and green emissions of the UCNPs to generate reactive oxygen species (ROS), respectively. The ROS generation in a dual photosensitizer system is significantly higher than that in a single photosensitizer system. Additionally, PDT induces immunogenic apoptosis. In this study, by utilizing a highly efficient PDT agent, PDT-induced apoptosis was studied by biomarker analysis.

## Introduction

Various anticancer therapies are currently being tested in clinics. However, new cancer remedies are still needed for selective and efficient treatment with minimal side effects. Photodynamic therapy (PDT) is one such method where photosensitizer molecules are excited by light to produce cytotoxic reactive oxygen species (ROS) to kill tumor cells (Dolmans et al., 2003). PDT is a minimally invasive therapeutic modality when compared to traditional surgical treatments. Light-induced local PDT treatment reduces the side effects of normal cell death by administering non-specific anticancer drugs. PDT is also known to induce an immune response by damaged tumor cells, which releases tumor-associated antigens and elicit antitumor immune responses (Maeding et al., 2016; Yang et al., 2016). PDT is usually used to treat local lesions by irradiation with light and the induction of immune responses opens the possibility of treating metastatic tumors or distant sites that cannot be reached by light (Zheng et al., 2016; Xu et al., 2017a; Hwang et al., 2018; Yan et al., 2019).

The PDT requires highly efficient photosensitizers to produce cytotoxic ROS. However, conventional photosensitizers used in PDT are usually excited by visible light, which limits the penetrating depth of the light source in tissues. Therefore, to increase the light penetration depth, it is necessary to develop efficient PDT agents that are excited by long wavelengths, such as near-infrared (NIR) light (Kobayashi and Choyke, 2019). Recently, lanthanide-doped upconversion nanoparticles (UCNPs) were developed to achieve NIR-triggered PDT (Guo et al., 2010; Tsai et al., 2018). As an anti-Stokes shifting material, UCNPs are the most efficient materials for converting NIR light to UV and visible light (Chen et al., 2014; Dong et al., 2015). Therefore, UCNPs can be used as a transducer to activate a conventional photosensitizer that is sensitive to visible light by using a NIR light source. Additionally, the use of NIR photons minimizes phototoxicity and background autofluorescence (Park et al., 2009; Wu et al., 2009), which is beneficial for bioimaging, diagnosis, and therapy. UCNP-based theranostic agents have been reported for simultaneous diagnosis and treatment of diseases (Park et al., 2012; Feng et al., 2019; Huang et al., 2020).

In this study, NIR-responsive PDT agents with dual photosensitizers were prepared. The 808 nm wavelength excitation was selected instead of the 980 nm wavelength to minimize overheating by the light source (Wang et al., 2013). For efficient photosensitizer activation using the 808 nm NIR wavelength excitation, NaYF_4_:Yb,Er,Nd@NaYF_4_:Yb,Nd was selected as Nd-doped UCNPs with a core@shell structure (Huang and Lin, 2015). The dual photosensitizers, Chlorin e6 and Rose Bengal, were used to activate the red and green emission of UCNPs. The dual photosensitizer system showed a synergistic ROS generation, as compared to the single photosensitizer system (Idris et al., 2012; Xu et al., 2017b; Yang et al., 2019). The ROS production achieved by NIR and PDT-induced apoptosis was investigated with regard to cellular studies.

## Experimental Section

### Synthesis of Photosensitizer-Loaded Upconversion Nanoparticles

#### Synthesis of NaYF_4_:Yb,Er,Nd Core UCNPs

The UCNPs were synthesized using a previously reported method with slight modifications (Huang and Lin, 2015). For NaYF_4_Yb,Er,Nd core UCNPs, 0.795 mmol of yttrium acetate hydrate (99.9%), 0.19 mmol of ytterbium acetate hydrate (99.9%), 0.005 mmol of erbium acetate hydrate (99.9%), and 0.01 mmol of neodymium acetate hydrate (99.9%) were added into a three-necked flask, and mixed with 11.84 g of oleic acid (OA, technical grade, 90%) and 5.32 g of 1-octadecene (ODE, technical grade, 90%). The residual water in the reaction mixture was removed at 150°C after 40 min. After cooling the temperature to 100°C, a methanol solution containing 0.1 g of NaOH (97%) and 0.148 g of NH_4_F (99.9%) was added to the flask and stirred for 20 min. The methanol was removed after 10 min at 110°C under vacuum. Then, the flask was heated to 310°C at a rate of 10°C/min in an Ar atmosphere and the temperature was maintained for 60 min. The core UCNPs were washed using ethanol and dispersed in cyclohexane.

#### Synthesis of NaYF_4_:Yb,Er,Nd@NaYF_4_:Yb,Nd corexs@shell UCNPs

The core@shell UCNPs were synthesized by the epitaxial growth of the shell on the core nanoparticle surface. For the NaYF_4_:Yb,Nd shell, 0.7 mmol of yttrium acetate hydrate, 0.1 mmol of ytterbium acetate hydrate, and 0.2 mmol of neodymium acetate hydrate were mixed in a three-necked flask with 11.84 g of OA and 5.32 g of ODE. After removing the residual water at 150°C, the flask was cooled to 70°C. A solution of core UCNPs in cyclohexane was added to the flask. The remaining synthetic procedure of the core@shell UCNPs was similar to that of the core UCNPs. The size and shape of the UCNPs were measured using transmission electron microscopy (TEM, JEOL-2100F, Japan), and the crystalline structures were recorded using an X-ray diffractometer (XRD, Rigaku, Rint 1000, Japan). The emission spectra of UCNPs were measured using a spectrophotometer (FluoroMax-4, Horiba) equipped with an 808 nm continuous wave (CW) laser (Opto Engine LLC). An inductively coupled plasma atomic emission spectrophotometer (ICP-AES) was used to determine the concentration of UCNPs.

#### Surface Modification of UCNPs

To impart water dispersibility, as-synthesized hydrophobic UCNPs were encapsulated with PEG-phospholipids (1,2-distearoyl-sn-glycero-3-phosphoethanolamine-N-[methoxy (po- lyethylene glycol)-2000], DSPE-PEG, Avanti Polar Lipids, Inc.) (Park et al., 2012). The UCNPs were mixed with excess DSPE-PEG in 10 mL of chloroform. After the complete removal of chloroform, the DSPE-PEG encapsulated UCNPs were redispersed in water. The excess DSPE-PEG was removed by repeated centrifugation (13,200 rpm for 20 min).

#### Loading Photosensitizers on the DSPE-PEG Encapsulated UCNPs

The DSPE-PEG encapsulated UCNPs were mixed with a photosensitizer solution containing 1 μmol of chlorin e6 (Ce6, Frontier Scientific, Inc.) or rose bengal (RB, Sigma-Aldrich). Depending on the photosensitizer loaded, four types of samples (e.g., UCNPs, RB-UCNPs, Ce6-UCNPs, and RB/Ce6-UCNPs) were prepared. The mixture was wrapped with an Al foil and incubated overnight at room temperature. The unloaded photosensitizers were removed by centrifugation, and the photosensitizers-loaded UCNPs (PS-UCNPs) were further purified using the desalting column (PD 10, GE Healthcare). The number of PS per UCNPs were quantified by measuring the absorbance of the PS in UCNPs (∼8 × 10^3^ Ce6 and ∼1 × 10^3^ RB per RB/Ce6-UCNPs).

### Measurement of ROS Generation From PS-UCNPs

A 10 μM stock solution of 9,10-anthracenediyl-bis(methylene)dimalonic acid (ABDA, Sigma-Aldrich) was prepared in water. Then, 1 mL of the ABDA stock solution was added to 1 mg of UCNPs loaded with different photosensitizers, respectively. The mixtures were irradiated using the 808 nm CW laser (500 mW, Opto Engine LLC). The decrease in fluorescence intensity of ABDA (λ_ex_ 380 nm and λ_em_ 407 nm) was measured using a spectrofluorometer (FluoroMax-4, Horiba).

### Cytotoxicity Assessment and Cellular PDT of PS-UCNPs

The B16BL6 melanoma cells (ATCC) were cultured in DMEM containing 10% FBS and 1% penicillin/streptomycin in 5% CO_2_ at 37°C. The cells were seeded in a 96-well microplate at a density of 5 × 10^3^ cells per well and incubated for 24 h (Xu et al., 2017a). The B16BL6 melanoma cells were treated with seven different concentrations (from 0.2 to 150 μg/mL) of UCNPs, RB-UCNPs, Ce6-UCNPs, and RB/Ce6-UCNPs for 24 h. The cytotoxicity of the samples was assessed by thiazolyl blue tetrazolium bromide (MTT, Sigma-Aldrich) cell proliferation assay. The MTT absorbance was measured using a spectrophotometer (Infinite 2000, Tecan) and IC_50_ was calculated using GraphPad Prism. To assess the cellular PDT effect, the cells with PS-UCNP uptake were irradiated for 10 min using the 808 nm CW laser (∼600 mW) and incubated for 24 h (Xu et al., 2017a; Ding et al., 2018). The laser beam was focused at for different points in the same well for 10 min to illuminate the whole cells as much as possible (approximately 2.5 min of irradiation for each point). The cell survival was assessed using the MTT assay.

### Detection of ROS From PS-UCNPs in Live Cells

The B16BL6 melanoma cells were seeded in a 96-well plate at a density of 5 × 10^3^ cells per well. The UCNPs, RB-UCNPs, Ce6-UCNPs, and RB/Ce6-UCNPs were incubated with the cells for 24 h, respectively. After irradiation by the 808 nm CW laser for 10 min, the cells were incubated for 24 h. The ROS in the live cells were measured using the DCFDA/H2DCFDA assay kit according to the manufacturer’s instructions (Abcam). The one-way ANOVA test was used for the statistical analysis of significance, followed by a Tukey’s *t*-test. *p*-values of ^∗∗∗^ < 0.001 were considered statistically significant.

### Fluorescence Imaging of ROS From PS-UCNPs in Live Cells

To confirm the ROS generation, *in vitro* cellular studies were performed on the B16BL6 melanoma cells. The B16BL6 melanoma cells (5 × 10^3^ cells per dish) were seeded in a 35 mm^2^ glass-bottom dish and incubated for 48 h. The cells were incubated with UCNPs, RB-UCNPs, Ce6-UCNPs, and RB/Ce6-UCNPs for 24 h, respectively, and irradiated using the 808 nm CW laser for 10 min. After 24 h, the cells were stained with CellROX deep red (5 μM, Invitrogen), CellMask Green (1:1000, Invitrogen), and Hoechst 33352 (1:5000, Invitrogen). The non-treated cells were used as control. The fluorescence images were obtained using an LSM 780 confocal microscope (Carl Zeiss 780) with a water immersion 40× lens.

### Western Blot of Cellular Proteins

The cellular protein expression was determined using western blot. The cellular protein was extracted using RIPA buffer (Abcam) and the concentrations were confirmed using the BCA assay kit (Thermo Fisher Scientific, Inc.). The electrophoresis was performed using 8% polyacrylamide gels (30 μg/lane). The gels were transferred to nitrocellulose membranes and incubated with a mouse monoclonal antibody against HSP70 (72 kDa, 1:1000, Invitrogen) and rabbit polyclonal antibody against HMGB1 (29 kDa, 1:500, Abcam), respectively. The mouse monoclonal antibody against GAPDH (36 kDa, 1:3000, Invitrogen) was used as the internal control. The signals were visualized using ECL and detected using the ImageQuant LAS 4000 system (GE Healthcare).

### Active Caspase-3 Colorimetric Assay

The cell apoptosis was measured using the caspase-3 assay kit (Abcam). The B16BL6 melanoma cells were seeded in a 96-well plate at a density of 5 × 10^3^ cells per well and the UCNPs, RB-UCNPs, Ce6-UCNPs, and RB/Ce6-UCNPs were incubated for 24 h, respectively. After irradiation with an 808 nm CW laser for 10 min, the cells were incubated for 24 h. The cells were lysed and centrifuged at 10,000 × *g*. The supernatant was collected and the assay was performed by following the manufacturer’s instructions (Abcam). The one-way ANOVA test was used for the statistical analysis of significance, followed by a Tukey’s *t*-test. *p*-values of ^∗∗∗^ < 0.001 were considered statistically significant.

## Results and Discussion

### Preparation and Characterization of PS-UCNPs

**FIGURE 1:**
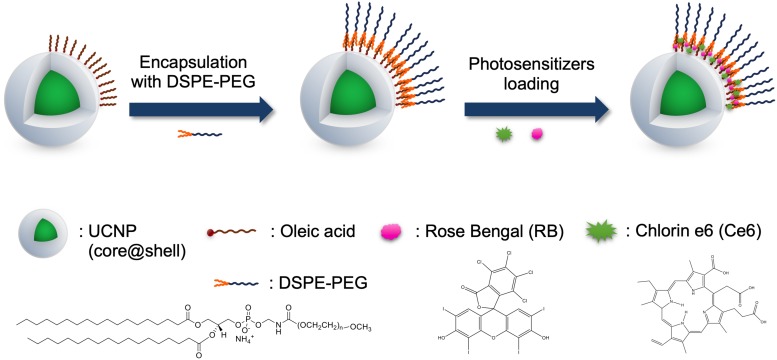
Schematic illustration of PS-UCNPs.

The overall synthetic procedure for preparing PS-UCNPs is illustrated in Figure 1. The highly crystalline NaYF_4_:Yb,Er,Nd@NaYF_4_:Yb,Nd core@shell UCNPs were synthesized using a high-temperature thermal decomposition method (Wang et al., 2010). The hydrophobic surface of the synthesized core@shell UCNPs was converted to hydrophilic by DSPE-PEG encapsulation (Park et al., 2012). DSPE-PEG is an amphiphilic consisting of a hydrophilic PEG chain and hydrophobic hydrocarbon chain. The hydrophobic UCNPs were encapsulated by DSPE-PEG through a hydrophobic interaction between the surface oleic acid and DSPE. The hydrophobic photosensitizers, Ce6 and RB, were also loaded in the hydrophobic layer (He et al., 2018).

To reduce the overheating effect of the 980 nm NIR light source, Nd ions were added to the core UCNPs with Yb ions as a sensitizer (Wang et al., 2013). The 808 nm NIR photons were absorbed by the Nd ions and transferred to the Yb ions for upconversion luminescence (Supplementary Figure S1). Continuous laser irradiation resulted in almost no increase in the temperature of the UCNP sample (Supplementary Figure S2). To improve the upconversion emission efficiency, the shell was grown on the core UCNPs to minimize non-radiative decay on the surface. Unlike the conventional inert shell without any sensitizers (e.g., NaYF_4_ or NaGdF_4_), the active shell including Yb and Nd ions was selected for more efficient 808 nm-triggered upconversion luminescence (Huang and Lin, 2015).

**FIGURE 2:**
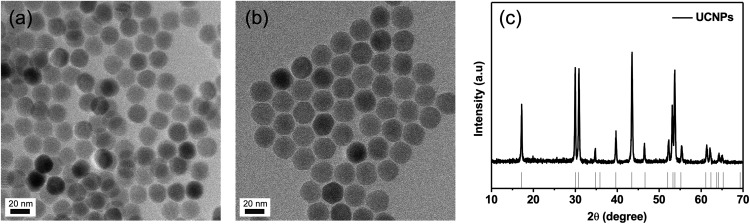
Characterization of UCNPs. **(a)** TEM image of core UCNPs, **(b)** TEM image of core@shell UCNPs, and **(c)** XRD patterns of core@shell UCNPs.

The synthesized NaYF_4_:Yb,Er,Nd core UCNPs were 18 nm in diameter with a uniform spherical shape (Figure 2a). After the epitaxial growth on the NaYF_4_:Yb,Nd shell of the core UCNPs, the core@shell UCNPs had a uniform size of 22 nm (Figure 2b). The uniform core@shell UCNPs with approximately 4 nm size increment demonstrated the successful synthesis of core@shell UCNPs. The XRD patterns of the UCNPs were well-matched with the hexagonal phase of the β-NaYF_4_ crystals (JCPDS No. 016-0334) (Figure 2c). The composition of the UCNPs was also measured using ICP-AES (Supplementary Table S1). The core and core@shell UCNPs had a similar molar ratio of the lanthanide elements to the theoretical values, indicating successful synthesis of core@shell UCNPs.

**FIGURE 3:**
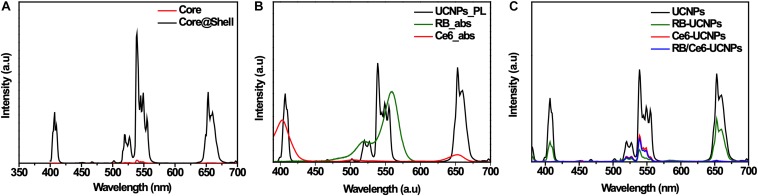
Optical properties of UCNPs and PS-UCNPs. **(A)** Photoluminescence (PL) spectra of core UCNPs and core@shell UCNPs. **(B)** Absorbance spectra of RB and Ce6, and PL spectrum of UCNPs. **(C)** PL spectra of four kinds of UCNP samples (Bare, RB-loaded, Ce6-loaded, and RB and Ce6-loaded samples).

The upconversion emission spectra of UCNPs were obtained with an 808 nm excitation (500 mW/cm^2^) (Figure 3A). The active shell growth enhanced the overall emission intensity by approximately 60 times. As shown in the elemental analysis (Supplementary Table S1), more Nd ions were doped into the shell to collect more 808 nm NIR photons, which may affect the significant increase in luminescence efficiency. The green emission at 520 nm (^2^H_11__/__2_^4^I_15__/__2_) and 539 nm (^4^S_3__/__2_^4^I_15__/__2_), the red emission at 653 nm (^4^F_9__/__2_^4^I_15__/__2_), and the blue emission at 407 nm (^2^H_9__/__2_^4^I_15__/__2_) were observed, respectively (Figure 3A and Supplementary Figure S1).

Chlorin e6 and RB were selected as the photosensitizers as their absorption wavelengths were well-matched with the emission wavelengths of UCNPs. Figure 3B shows that the absorption of Ce6 overlaps with the blue and red emission of UCNPs, and that of RB overlaps with the green emission of UCNPs. Regardless of the type of photosensitizers, the upconversion emission of PS-UCNPs was significantly reduced after photosensitizer loading (Figure 3C and Supplementary Figure S3). The red and blue emission shows an obvious decrease for Ce6-UCNPs, while the green emission significantly decreased for RB-UCNPs. This indicates that the emissions of the UCNPs have been absorbed by the photosensitizers, and the energy transfer between the photosensitizers and UCNPs was successful.

**FIGURE 4:**
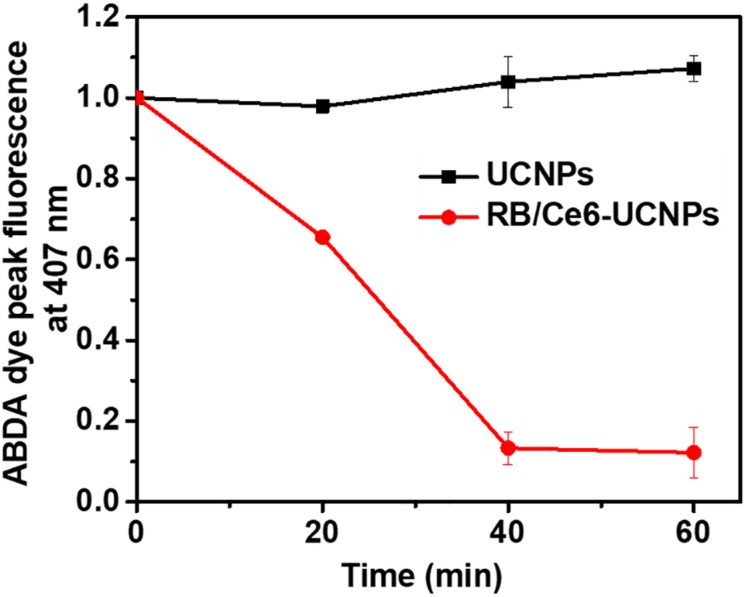
Measurement of ROS generation by RB and Ce6-loaded UCNPs under 808 nm NIR irradiation. PL intensities of ABDA dye were measured at 407 nm.

The production of ROS by PS-UCNPs was evaluated using ABDA (Lindig et al., 1980; Shen et al., 2011). ABDA selectively reacts with singlet oxygen and decomposes to decrease the fluorescence. Compared to the control sample without a photosensitizer, the fluorescence intensity in each sample decreases (Supplementary Figure S3). This means that ROS were generated by the 808 nm NIR excitation. Among them, the fluorescence intensity in RB/Ce6-UCNPs significantly decreases, indicate that UCNPs with dual photosensitizers were more efficient in ROS production and have improved PDT efficiency (Figure 4).

### Cytotoxicity and Cellular PDT Effect of PS-UCNPs

**FIGURE 5:**
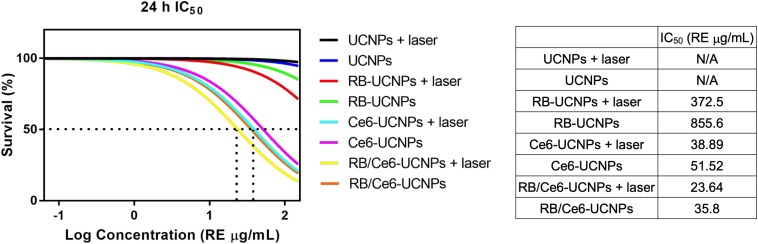
Cytotoxicity of UCNPs and PS-UCNPs in B16BL6 melanoma cells with or without 808 nm laser irradiation. The IC_50_ values after 24 h of treatment were measured. The survivals were determined by the MTT assay using three replicated samples from three separate experiments. Results are presented as mean ± SEM.

The cytotoxic effects of various treatments were evaluated in B16BL6 mouse melanoma cells using the MTT assay. A dosage of 5.6 μg/mL of RB/Ce6-UCNPs showed a 92% survival rate for 24 h incubation. However, the survival rate significantly decreased to 30% at 50 μg/mL of RB/Ce6-UCNPs (Supplementary Figure S4a). Compared to RB/Ce6-UCNPs, the UCNP and RB-UCNP treatments had no significant effect on the cell survival rate up to 100 μg/mL (Figure 5 and Supplementary Figure S5). The cytotoxicity of RB/Ce6-UCNPs without laser irradiation seems to be due to Ce6 released from the UCNPs. Ce6-UCNPs also showed a similar cytotoxic effect as RB/Ce6-UCNPs. When the B16BL6 melanoma cells were treated with RB/Ce6-UCNPs and 808 nm CW laser irradiation for 10 min, the cell viability was further decreased to 84% at 5.6 μg/mL and 24% at 50 μg/mL (Supplementary Figure S4b). Figure 5 shows that the laser irradiation resulted in lower IC_50_ values than non-laser irradiated treatment regardless of the combination of the photosensitizers. Among them, the RB/Ce6-UCNPs with laser irradiation killed the B16BL6 cells more effectively than single photosensitizer-loaded UCNPs. Accordingly, 16.7 μg/mL of RB/Ce6-UCNPs and 10 min irradiation were selected as the conditions for the following PDT experiments without significant cytotoxicity of the photosensitizer itself.

### PDT-Induced Intracellular ROS Generation and Apoptosis

**FIGURE 6:**
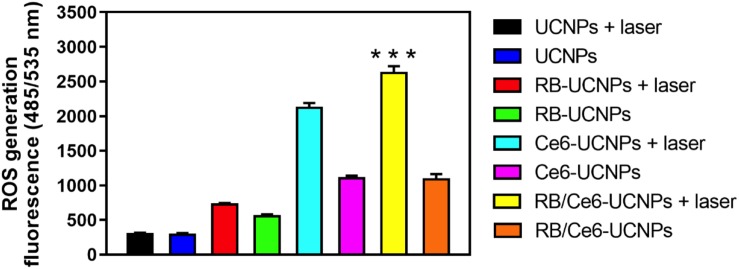
Quantified ROS generation under laser irradiation in B16BL6 melanoma cells. The cellular ROS was measured using the DFCDA/H2DCFDA kit. The cells were incubated with each UCNP samples (100 RE μg/mL) for 3 h and then irradiated using an 808 nm NIR laser for 10 min. All experiments were performed in triplication. The results are presented as mean ± SEM. ***p < 0.001.

**FIGURE 7:**
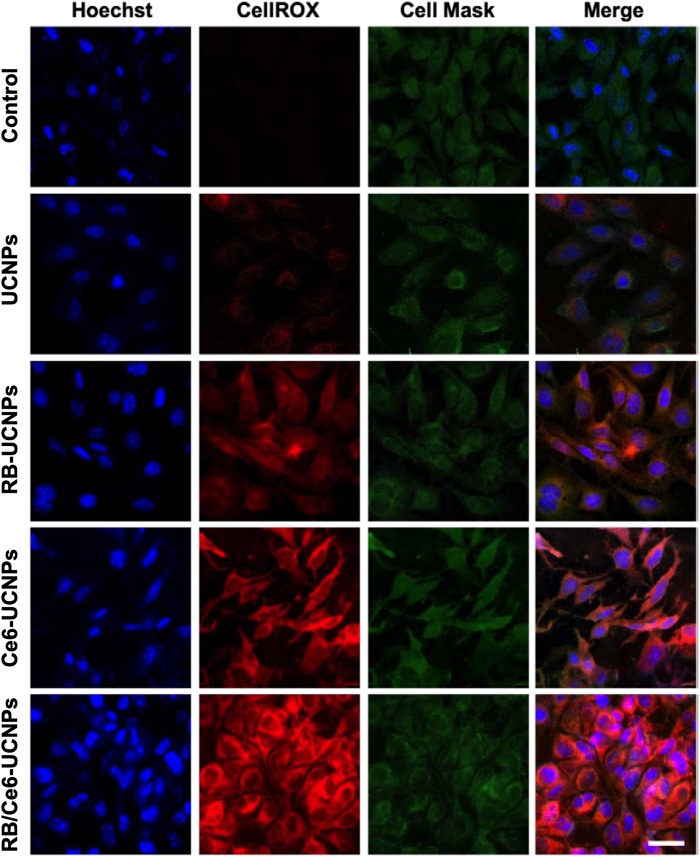
Representative confocal images of laser mediated ROS generation in B16BL6 melanoma cells. The cells were incubated with each UCNP samples (100 RE μg/mL) for 3 h and then irradiated using an 808 nm NIR CW laser for 10 min. All experiments were performed in triplication. The scale bar is 25 μm.

The activated photosensitizer under laser irradiation can generate ROS, which can destroy the tumor cells through the apoptosis or necrosis process (Yang and Wang, 2013). The ability of UCNPs to generate ROS was evaluated using the DCFDA/H2DCFDA assay kit in B16BL6 melanoma cells. As shown in Figure 6, the 808 nm laser irradiation allows PS-UCNPs to increase ROS generation than that without laser irradiation. In particular, the laser-irradiated RB/Ce6-UCNPs showed significantly higher ROS generation in the experimental group. Similar to the DCFDA/H2DCFDA assay, the intracellular ROS generation by PS-UCNPs was confirmed using a fluorogenic probe, CellRox deep red, in live B16BL6 melanoma cell imaging (Figure 7). The deep red signal by RB/Ce6-UCNPs was much stronger than that treated with RB-UCNPs or Ce6-UCNPs, indicating a very efficient ROS generation in cells.

**FIGURE 8:**
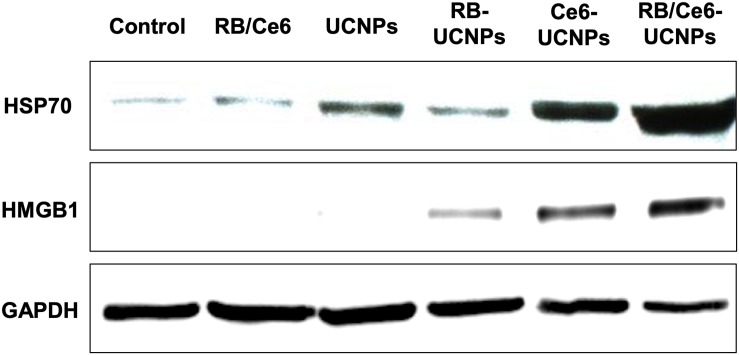
Cellular protein expression of HSP70 (70 kDa) and HMGB1 (29 kDa). The B16BL6 melanoma cells were incubated with each UCNP samples (100 RE μg/mL) for 3 h and then irradiated using an 808 nm NIR CW laser for 10 min. Ten spots were selected and irradiated in each well. GAPDH (38 kDa) was used as an internal control. All experiments were performed in triplication.

The PDT-induced oxidative stress resulted in extended tumor apoptosis (Yang et al., 2016). The damaged or dying tumor cells can be characterized by detecting damage-associated molecular patterns (DAMPs), such as heat shock protein 70 (HSP70) (Korbelik et al., 2005) and high mobility group box 1 (HMGB1) (Panzarini et al., 2013). In particular, HSPs have been known to inhabit in all blood cells and migrate to the cell surface membrane during apoptosis induced by PDT (Zhou et al., 2008; Yang et al., 2016). The expression of HSP70 and HMGB1 protein by PDT was confirmed by western blot. The laser-irradiated RB/Ce6-UCNPs significantly increased the expression levels of HSP70 and HMGB1 protein in B16BL6 melanoma cells (Figure 8).

**FIGURE 9:**
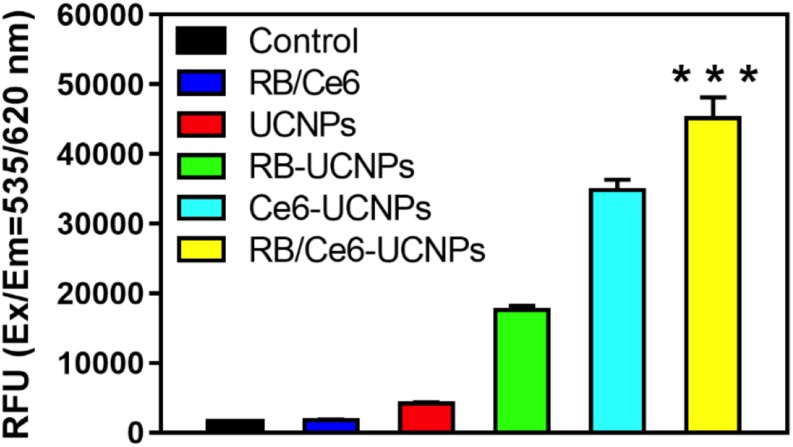
Cellular caspase-3 activity was measured using the fluorometric kit. The B16BL6 melanoma cells were cultured with each UCNP samples (100 RE μg/mL) for 3 h and irradiated using an 808 nm NIR CW laser for 10 min. Ten spots were selected and irradiated in each well. The extracted cell proteins were confirmed according to the manufacturer’s instruction. All experiments were performed in triplication. The results are presented as mean ± SEM. ***p < 0.001.

The PDT can induce apoptosis or necrosis, or a combination thereof, although PDT is highly efficient at inducing apoptosis mainly (Oleinick and Evans, 1998). This study investigated the active caspase-3 downstream apoptotic marker using a colorimetric assay kit. Figure 9 shows a significant increase in caspase-3 activity when a laser is irradiated to RB/Ce6-UCNPs treated cells. The RB/Ce6-UCNPs showed activated caspase-3 levels of 1.3 and 2.7 times higher than Ce6-UCNPs and RB-UCNPs, respectively. These results demonstrate that dual PS-UCNPs show high efficacy and a synergistic effect in PDT therapy.

## Conclusion

In this study, UCNPs with dual photosensitizers were developed as a highly efficient NIR-triggered PDT agent. An 808 nm excitation wavelength was used to minimize overheating by the NIR light source. As the addition of the energy transfer step of UCNPs by Nd ions reduces the upconversion efficiency, a core@shell structure with an active shell was prepared. The use of dual photosensitizers for the red and green emission of UCNPs improved the ROS generation efficiency. A synergistic ROS generation of PS-UCNPs was studied in cellular studies and the PDT-induced apoptosis was confirmed. However, the leakage of PS from the UCNPs, in particular Ce6, seems to induce cytotoxicity. Therefore, it is necessary to improve the PS loading method to minimize PS leakage and ensure long term stability.

## Data Availability Statement

All datasets generated for this study are included in the article/Supplementary Material.

## Author Contributions

SYL, RL, SL, and YP designed the research and wrote the manuscript with inputs from EK. SYL synthesized and characterized the PS-UCNPs under the guidance of YP. RL, EK, and SL performed the cell experiments.

## Conflict of Interest

The authors declare that the research was conducted in the absence of any commercial or financial relationships that could be construed as a potential conflict of interest.
