# Supplementary material for: Near-Infrared Light-Triggered Photodynamic Therapy and Apoptosis Using Upconversion Nanoparticles With Dual Photosensitizers
Source: Front Bioeng Biotechnol. 2020 Apr 16;8:275. doi: 10.3389/fbioe.2020.00275 (PMC7179334; doi:10.3389/fbioe.2020.00275)
Supplement: Supplementary file 1 [file Data_Sheet_1.pdf]

Supplementary Material

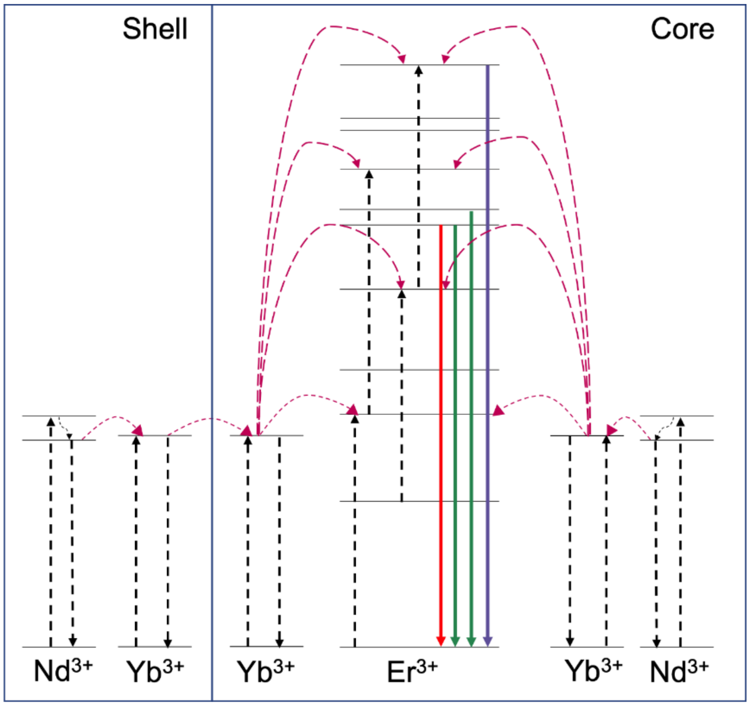

Supplementary Figure 1. Schematic diagram of upconversion processes of Nd-doped UCNPs with a core@shell structure.

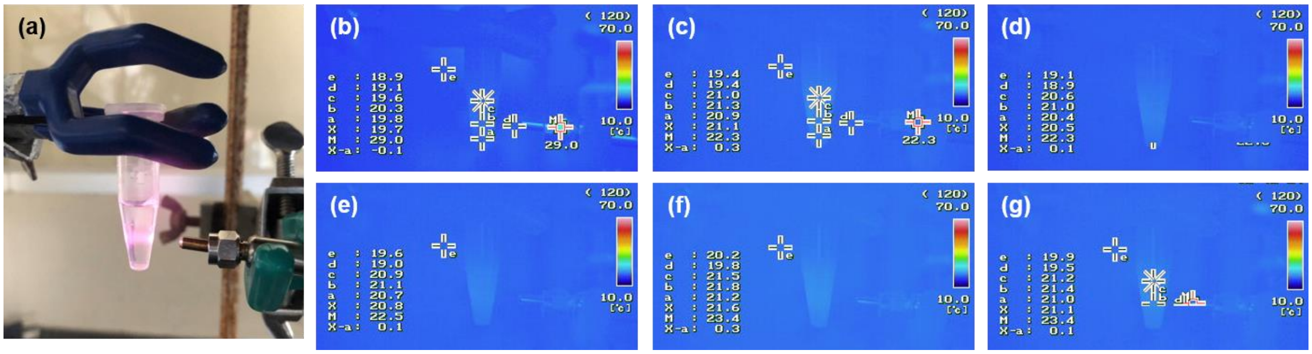

Supplementary Figure 2. Measurement of the heating effect by an 808 nm CW laser. (a) Photograph of UCNP sample irradiated with an 808 nm CW laser (1 W). (b-g) Thermal images of UCNP samples at different irradiation time; (b) 0 min, (c) 10 min, (d) 20 min, (e) 30 min, (f) 40 min, (g) 50 min. In the thermal images, the b position represents the position through which the laser beam passes, and a and c represent positions below and above the laser beam, respectively.

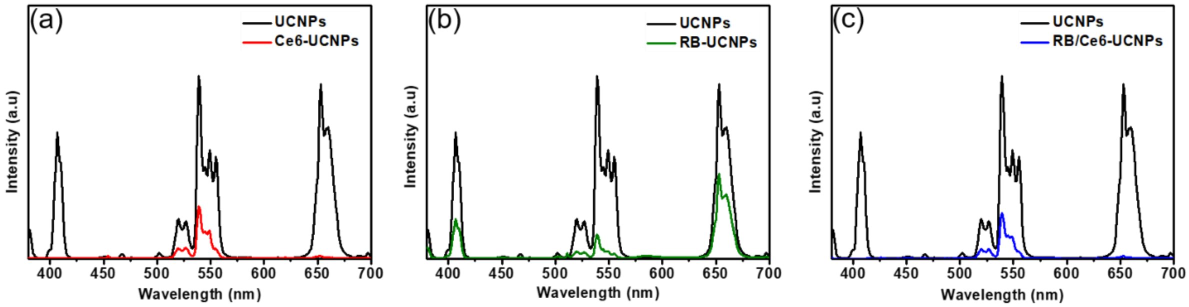

Supplementary Figure 3. PL spectra of UCNPs and PS-UCNPs. (a) Ce6-loaded, (b) RB-loaded, and (c) RB and Ce6-loaded UCNPs.

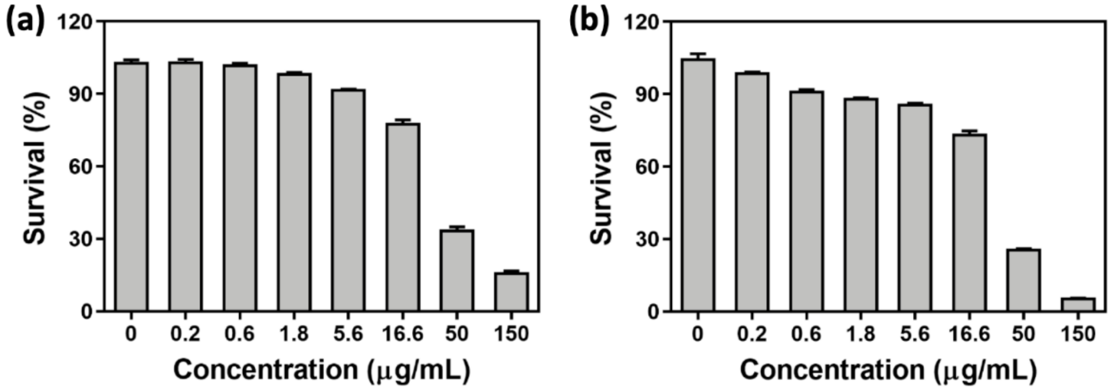

Supplementary Figure 4. Cytotoxicity of RB and Ce6-loaded UCNPs in B16BL6 melanoma cells (a) without and (b) with 808 nm laser irradiation.

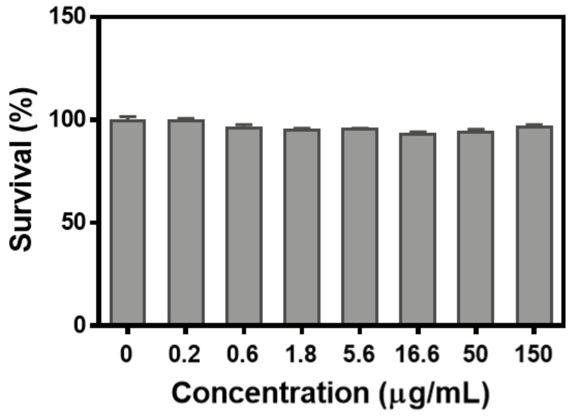

Supplementary Figure 5. Cytotoxicity of UCNPs in B16BL6 melanoma cells.

Supplementary Table 1. Elemental analysis of UCNPs.

	Y (mol%)	Yb (mol%)	Er (mol%)	Nd (mol%)
Core UCNPs	78.4	20.4	0.4	0.8
Core/Shell UCNPs	76.6	14.0	0.3	9.1
